# Interleukin-7-aggravated joint inflammation and tissue destruction in collagen-induced arthritis is associated with T-cell and B-cell activation

**DOI:** 10.1186/ar3870

**Published:** 2012-06-07

**Authors:** Sarita AY Hartgring, Cynthia R Willis, Johannes WJ Bijlsma, Floris PJG Lafeber, Joel AG van Roon

**Affiliations:** 1 Department of Rheumatology & Clinical Immunology, UMC Utrecht, Heidelberglaan 100, Utrecht, PO Box 85500 F02.127 3508 GA, The Netherlands; 2 Inflammation Department, Amgen Inc., 1201 Amgen Court West, Seattle, WA 98119, USA

## Abstract

**Introduction:**

We sought to investigate the capacity of interleukin (IL)-7 to enhance collagen-induced arthritis and to study by what mechanisms this is achieved.

**Methods:**

Mice received multiple injections with IL-7 or phosphate-buffered saline (PBS) as a control. Arthritis severity and incidence were determined by visual examination of the paws. Joint destruction was determined by assessing radiographs and immunohistochemistry of the ankle joints. Total cellularity and numbers of T-cell and B-cell subsets were assessed, as well as *ex vivo *production of interferon-γ (IFN-γ), IL-17, and IL-4. Proinflammatory mediators were measured in serum with multianalyte profiling.

**Results:**

IL-7 increased arthritis severity and radiology-assessed joint destruction. This was consistent with IL-7-increased intensity of cell infiltrates, bone erosions, and cartilage damage. Splenic CD19^+ ^B cells and CD19^+^/GL7^+ ^germinal center B cells, as well as CD4 and CD8 numbers, were increased by IL-7. IL-7 expanded memory T cells, associated with increased percentages of IFN-γ-, IL-4-, and IL-17-producing CD4^+ ^T cells. On antigen restimulation of draining lymph node cells *in vitro *IL-7 treatment was found to increase IFN-γ and IL-17 production, whereas IL-4 was reduced. IL-7 also increased concentrations of proinflammatory mediators, indicative of T-cell activation (sCD40L), vascular activation (VCAM-1, VEGF), tissue destruction (fibroblast growth factor-basic (FGF-b), LIF), and chemotaxis (MIP-1γ, MIP-3β, lymphotactin, MDC, and MCP-5).

**Conclusions:**

In arthritic mice, IL-7 causes expansion of T and B cells, associated with increased levels of proinflammatory mediators. IL-7 intensifies arthritis severity and joint destruction, accompanied by increased Th1 and Th17 activity. These data indicate that IL-7 could be an important mediator in arthritic conditions and that targeting IL-7 or its receptor represent novel therapeutic strategies.

## Introduction

Interleukin-7 (IL-7) is an immunostimulatory cytokine produced by stromal cells and plays a pivotal role in T-cell development in mice and humans [1,2]. B-cell development in mice is dependent on IL-7, but in humans, this is regulated differently [3]. IL-7R-deficient humans have reduced T-cell numbers, but not B-cell numbers. Reduced B-cell activity (immunoglobulin (Ig) levels) in IL-7R-deficient humans is therefore suggested to be T-cell driven [4]. IL-7 induces T-cell-dependent activation of monocytes and osteoclasts [1,2,5]. IL-7 in ovariectomized mice induces T-cell-mediated and receptor activator of nuclear factor (NF)-κB ligand (RANKL)- and tumor necrosis factor (TNF)-α-dependent generalized bone loss in the absence of inflammation [6].

High levels of IL-7 are found in several arthritic conditions, including rheumatoid arthritis (RA). Serum IL-7 levels in arthritic individuals are increased and correlate with markers of disease activity [7-9]. IL-7 levels in synovial fluid (SF) are also increased in RA. In RA synovial tissue, IL-7 is abundantly expressed by macrophages, endothelial cells, and fibroblasts, and IL-7 correlates with numbers of CD68^+ ^macrophages [10]. In arthritic individuals, IL-7 levels correlate with TNF-α [9]. Importantly, in RA patients that do not respond to anti-TNF-a treatment, IL-7 levels persist, indicating a role for IL-7, possibly independent of TNF-a, in immunopathology in specific groups of RA patients [9].

IL-7 effects are mediated through the IL-7 receptor-α chain (IL-7Rα) in conjunction with the common γ (gamma) chain. Intraarticular IL-7R expression is increased in the synovium of RA patients, and intraarticular numbers of IL-7R^+ ^cells correlate with CD3^+ ^T-cell counts and IL-7 expression. Furthermore, the IL-7R is present on highly proliferating synovial T cells but not on regulatory FoxP3^+ ^T cells [11].

Apart from the expression of IL-7 and IL-7R, the immunostimulatory capacities of IL-7 suggest an important contribution of IL-7 in joint inflammation in RA. IL-7 induces mainly T-cell activation but can also directly induce proinflammatory activities from several other cell types. IL-7-stimulated mononuclear cells from RA peripheral blood (PB) and SF produce primarily Th1 and Th17 cytokines [8,12], and IL-7 increases TNF-a and IFN-g production by RA PB T cells [13]. Additionally, IL-7 stimulates T-cell-dependent expression of co-stimulatory molecules on monocytes/macrophages, resulting in contact-dependent activation of T cells [9,10]. T cell-dependent activation of monocytes/macrophages by IL-7 is also associated with TNF-α production from monocytes [9,10]. Furthermore, IL-7 can directly stimulate monocytes to produce a number of proinflammatory cytokines (IL-1a, IL-1b, IL-6, IL-8, MIP-1b) [14-16]. Together, this indicates the importance of IL-7 in promoting inflammation and tissue destruction in RA.

Blocking IL-7 prevents gp-130-dependent autoimmune arthritis in mice [17]. Thymic stromal lymphopoietin (TSLP), an IL-7-related cytokine, also signals through the IL-7R (in conjunction with the TSLPR) and recently was shown to have arthritogenic potential [18]. In addition, IL-7R blockade prevents collagen-induced arthritis [19]. Although in this latter study, the used anti-IL7R antibody *in vitro *had a 100-fold stronger capacity to block IL-7-, as compared with blocking TSLP-induced signaling; this antibody potentially could also block IL-7-related TSLP. Therefore, we studied whether IL-7 in fully immunocompetent mice causes enhancement of experimental arthritis and by which mechanisms, specific for IL-7, this is mediated.

## Materials and methods

### Induction, treatment, and assessment of collagen-induced arthritis

Collagen-induced arthritis (CIA) was produced in 8-week old male DBA/1 mice (Harlan Laboratories, Inc., Indianapolis, IN, USA). In brief, on day 0, intradermal immunization injections were given at the base of the tail with chicken collagen type II (CII, Sigma C9301; St. Louis, MO, USA) dissolved in complete Freund adjuvant, followed by an intradermal booster injection of CII dissolved in incomplete Freund adjuvant (IFA) on day 21. Mice were examined for onset and severity of disease in a blinded manner, by using a scoring system (0 to 4), as previously described [19]. Each limb was graded, giving a maximum possible score of 16 per mouse. Arthritis incidence was defined by a single-paw score of ≥1. Recombinant mouse IL-7 (10 μg; R&D Systems, Inc., Minneapolis, MN, USA), or PBS were given intraperitoneally (IP) at the time of the boost (experimental day 21), day 23, day 25, day 27, day 29, and day 31. All mice were killed on experimental day 33. All experiments were performed in accordance with federal guidelines and approved by the Amgen Institutional Animal Care and Use Committee (IACUC).

### Assessment of radiologic and histologic joint damage in CIA

Ankle joints were isolated, fixed in formalin (24 hours), and lateral-medial radiographs were made. All ankles were scored for severity of radiographic lesions, according to a scoring index from 0 to 3 (previously described in [19]). Thereafter, ankles were decalcified in 10% ethylenediaminetetraacetic acid (EDTA) and paraffin embedded. Tissue sections (5 μm) were stained with hematoxylin-eosin (HE) and for cathepsin-K. Deparaffinized tissue sections were pretreated with 0.1% trypsin in 0.1% CaCl_2_, blocked with CAS-BLOCK (Zymed Laboratories, San Francisco, CA, USA), and incubated with rabbit polyclonal anti-cathepsin-K (Amgen Inc., Thousand Oaks, CA, USA). Slides were quenched with peroxidase blocking solution (Dako Corp., Carpenteria, CA, USA) and detected with Envision+System-HRP Labeled Polymer anti-rabbit (Dako). Reaction sites were visualized with DAB+Substrate-Chromagen System (Dako). Slides were scored as described previously [18]. The radiologic and histologic scores of two ankles were averaged per mouse, and 15 mice per group were compared for statistical analysis.

### Spleen and thymus cell preparation and flow cytometry

To prepare single-cell suspensions, spleen and thymus were pressed through a 70-μm cell strainer (Falcon; BD Biosciences, San Jose, CA, USA) into Hanks' balanced salt solution (HBSS; Invitrogen, Carlsbad, CA, USA) with 5% fetal bovine serum (FBS; Invitrogen). Red blood cells were lysed with ACK lysis buffer (Invitrogen). Cells were counted, pelleted, and resuspended in HBSS with 5% FBS and 1 µg/ml 2.4G2 (CD32/16; Fc-block, BD). For flow-cytometric analysis, monoclonal antibodies were used: anti-CD4 allophycocyanin (APC)/Cy7 (clone-Gk1.5; BD), anti-CD8 Pacific Blue (PB) (clone-53-6.7; BD), anti-CD19 peridinin-chlorophyll-protein (PerCP)/Cy5.5 (clone-1D3; BD), anti-CD44 FITC (clone-IM7; eBioscience, San Diego, CA, USA), and anti-CD62L APC (clone-mel-14; BD).

Before intracellular cytokine staining, splenocytes were restimulated with phorbol-12-myristate-13-acetate (PMA)/Ionomycin/BrefeldinA (Sigma) (final concentration, 50 ng/500 ng/10 μg/ml) for 4 hours, permeabilized with cytofix/cytoperm kit (BD), and stained with: anti-IFN-γ FITC (clone-XNG1.2; BD), anti-IL-4 APC (clone-11B11; BD), and anti-IL-17 phycoerythrin (PE) (clone-TC11-18H10; BD). Intracellular cytokine stainings were done for five of 15 mice per group with representative clinical arthritis severity; 10^5 ^events were collected by using FACS-LSRII (BD), and analyzed by using FlowJo software (Tree Star, Inc., Ashland, OR, USA).

### Detection of immunomodulatory proteins

Concentrations of specific proteins were assayed with Rules Based Medicine Inc. (Austin, TX) for rodent antigens (version 2.0) multianalyte-profiling (MAP), in serum samples from day 33.

### *In vitro *restimulation of lymph node cells

Cells were isolated from axial-, brachial-, and inguinal-draining lymph nodes (LNs) and crushed through a 70-μm cell strainer into HBSS with 5% FBS. Cells were cultured (1.10^6^/well) for 3 days with or without collagen type II (20 and 100 μg/ml). IFN (BD), IL-4 (BD), and IL-17 (R&D systems) concentrations in supernatants were measured with enzyme-linked immunosorbent assay (ELISA).

### Statistical analysis

Arthritis severity and numbers of splenocytes and splenocyte subsets were analyzed with an independent sample *t *test. Radiologic damage, numbers of thymocytes and thymocyte subsets, and cytokine concentrations in supernatants were analyzed by using an independent-sample *t *test. ELISA results were analyzed with a Kruskal-Wallis test followed by a Mann-Whitney *U *test for nonparametric distributed data. MAP data from serum samples were analyzed with one-way ANOVA after an independent-sample *t *test.

## Results

### IL-7 enhances severity of arthritis and arthritis-induced joint destruction

**Figure 1:**
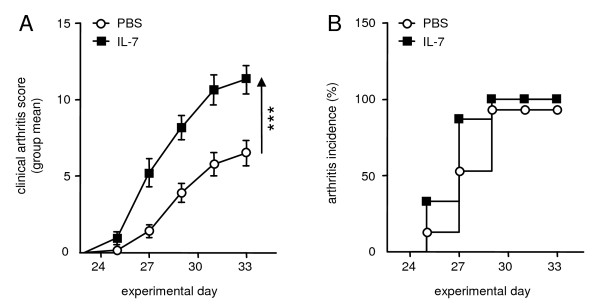
**Interleukin (IL)-7 enhances the severity of collagen-induced arthritis**. Mice were injected with either phosphate-buffered saline (PBS) or IL-7, every other day starting at day 21 and continuing until day 33, and arthritis severity was graded. IL-7 significantly increased arthritis severity compared with that in PBS-treated mice (on average from days 27 to 33 by 135% ± 46% **(A)**. IL-7 did not significantly change arthritis incidence **(B)**. Values are expressed as mean ± SEM of 15 mice per group. ***Statistical difference of *P *< 0.001 for area under the curve (A).

Mice were treated with IL-7 or PBS as a control to examine the effects of IL-7 on development of collagen-induced arthritis (CIA). IL-7 significantly increased arthritis severity by 135% ± 46% (days 27 through 33, mean ± SEM) compared with PBS-treated mice. On day 33, IL-7-treated mice had significantly higher (*P *< 0.001) mean clinical arthritis scores (11.3 ± 0.9) compared with PBS-treated mice (mean score ± SEM; 6.5 ± 0.8). Over the entire period, statistically significant higher arthritis scores for the IL-7-treated mice compared with the PBS-treated mice were found (mean area under the curve ± SEM; 61.2 ± 6.5 and 28.9 ± 5.2; *P *< 0.001, Figure 1A). At day 33, arthritis developed in 93% of the PBS-treated mice (14 of 15) compared with 100% in IL-7-treated mice (Figure 1B).

**Figure 2:**
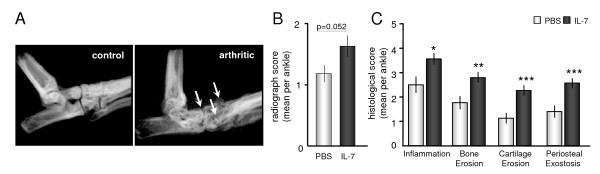
**Interleukin (IL)-7 increases joint damage and inflammation in collagen-induced arthritis**. Mice were injected with either phosphate-buffered saline (PBS) or IL-7. Radiographs of the ankle joints were taken at day 33. Radiologic joint damage was graded for each ankle with a scoring index from 0 to 3. **(A) **Representative radiographs show an unaffected control (nonarthritic) ankle joint, and space narrowing, variability in bone density, and roughened bone surface in an arthritic ankle joint (arrows). IL-7 significantly increased radiologic damage as compared with that in the PBS group (mean scores of 1.2 ± 0.1 versus 1.6 ± 0.2, respectively **(B)**. Consistent with the radiograph scores, histologic examination of the ankles showed significantly increased severity of inflammation, bone and cartilage erosions, and periosteal exostosis with IL-7 treatment compared with PBS **(C)**. Values are expressed as mean ± SEM of 15 mice per group (scores of two ankles per mouse were averaged); statistical differences with PBS group of *P < 0.05, **P < 0.01, and ***P < 0.005.

In both groups, radiographs from ankle joints showed joint-space narrowing, variability in bone density, roughened bone surface, and some mice showed osteophyte formation (indicated with arrows on representative radiographs; Figure 2A). Compared with the PBS group (mean score ± SEM, 1.2 ± 0.1), a significantly higher radiologic score (*P *= 0.052) was found for the IL-7 group (1.6 ± 0.2; Figure 2B). In line with this, histopathologic analysis revealed significantly increased inflammation (*P *< 0.05), subchondral bone erosion (*P *< 0.01), and articular cartilage erosion (*P *< 0.005) by IL-7 as compared with PBS (Figure 2C).

**Figure 3:**
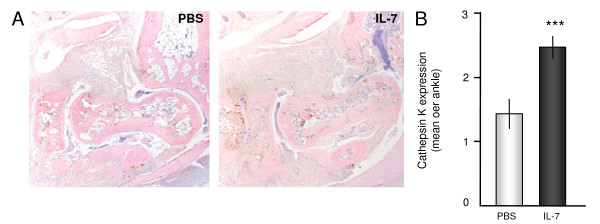
**Interleukin (IL)-7 increases osteoclast activity in collagen-induced arthritis**. Mice were injected with either phosphate-buffered saline (PBS) or IL-7. Osteoclast activity indicated by cathepsin K expression (brown) was graded for each ankle with a scoring index from 0 to 3. Representative cathepsin K staining with hematoxylin & eosin (H&E) counterstains from arthritic mice of each treatment group demonstrate bone erosion associated with high numbers of active osteoclasts (**A**; ×40 magnification of tibial tarsal joints). Osteoclast activity, indicated by cathepsin K expression, was significantly increased in IL-7-treated mice as compared with PBS-treated mice **(B) **(1.4 ± 0.2 versus 2.4 ± 0.2). Values are expressed as mean ± SEM of 15 mice per group. ***Statistical differences with PBS group of *P *< 0.005.

Because IL-7 induces osteoclast activation [20], we determined osteoclast activity, indicated by cathepsin-K expression (representative staining, Figure 3A). In line with bone erosion and radiographic damage, IL-7 administration significantly enhanced cathepsin-K expression (*P *= 0.003; Figure 3B).

### IL-7 expands T cells and B cells

**Figure 4:**
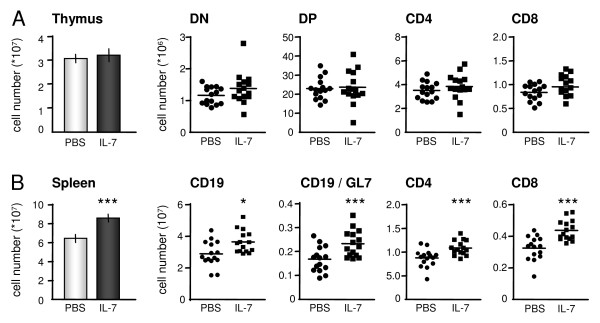
**Interleukin (IL)-7 promotes expansion of splenic T and B cells**. Total number of thymocytes was not affected by IL-7. The absolute numbers of CD4CD8 double negative (DN), CD4CD8 double positive (DP), and single-positive CD4 and CD8 thymocytes were not changed by treatment with IL-7 **(A)**. Peripheral cell numbers, measured by total numbers of splenocytes, CD4^+ ^T cells, CD8^+ ^T cells, CD19^+ ^B cells, and germinal center (GL7^+^) B cells were all increased by IL-7 compared with PBS treatment **(B)**. Values are expressed as mean ± SEM of 15 mice per group. Statistical differences with PBS group of *P < 0.05 or **P < 0.005.

IL-7 affects T- and B-cell expansion [1-5]; therefore, numbers of T and B cells were analyzed. Thymic output, indicated by absolute cell number, was not affected by IL-7 (Figure 4A). Also the tissue weight of the thymuses was not different between the PBS and IL-7 groups. Absolute numbers of CD4CD8 double-negative (DN), CD4CD8 double-positive (DP), and single-positive CD4 and CD8 thymocytes were not changed by IL-7 treatment (Figure 4A). Peripheral cell numbers, measured by total number of splenocytes, CD4^+ ^T cells, CD8^+ ^T cells, CD19^+ ^B cells, and germinal center (GL7^+^) B cells were all increased by IL-7 (Figure 4B).

**Figure 5:**
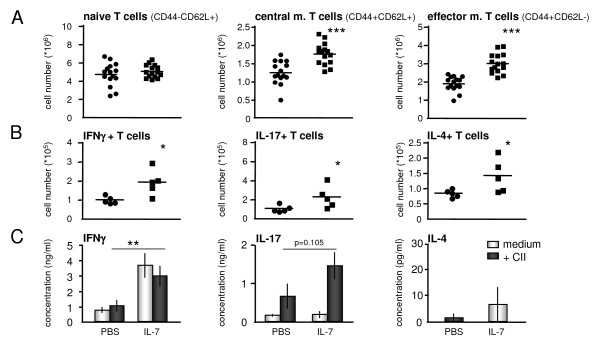
**Interleukin (IL)-7 promotes differentiation toward memory and effector T cells and cytokine-secreting T-helper cells**. The number of naive CD4 T cells (CD44-CD62L^+^) was not significantly altered by IL-7. By contrast, a significant increase in central memory (CD44^+^CD62L^+^) and effector memory (CD44^+^CD62L^-^) T cells was found after IL-7 administration **(A)**. IL-7 enhances numbers (and percentages) of CD4 T cells, and of Th1 (interferon (IFN)-g)-, and Th17 (IL-17)-, and Th2 (IL-4)-cytokine-secreting CD4 T cells **(B)**. Increased IFN-g was measured in supernatants from lymph node cells (LNs) of the IL-7-treated mice as compared with those of the PBS-treated mice. Moreover, on collagen type II (CII) restimulation of LNs, increased IL-17 and reduced IL-4 concentrations were measured, as compared with those of PBS-treated mice **(C)**. Statistical differences with PBS group of *P < 0.05, **P < 0.01, and ***P < 0.001.

Because IL-7 is also known to stimulate naive and memory T cells and to induce proinflammatory T-cell cytokine production, the effect on these cell subsets was studied. Naive CD4^+ ^T-cell (CD44-CD62L^+^) numbers were not significantly altered by IL-7 compared with PBS treatment (Figure 5A). By contrast, a significant increase in central memory (CD44^+^CD62L^+^) and effector memory (CD44^+^CD62L^-^) T cells was found after IL-7 treatment (*P *< 0.001 for IL-7 as compared with PBS). In addition, numbers of splenic T cells producing IFN-γ (Th1), IL-17 (Th17), and IL-4 (Th2) were significantly increased in IL-7-treated mice as compared with PBS (both *P *< 0.05; Figure 5B).

To study the IL-7 effect on local antigen-driven responses, draining lymph node cells (LNs) were restimulated *in vitro *with type II collagen (CII), and IFN-γ, IL-17, and IL-4 concentrations in supernatants were measured. CII-restimulated LN cells from PBS-treated mice showed increased IFN-γ production (mean concentration ± SEM; 1,0 ± 0,3 ng/ml) compared with unstimulated cells (medium only; 0,7 ± 0,2 ng/ml). IL-7 treated mice showed strongly increased IFN-γ production by LN cells (on average, threefold higher as compared with the PBS-treated mice). In IL-7-treated mice, restimulation with CII did not further increase this increased IFN-γ production (unstimulated versus CII stimulated; 3,6 ± 0,8 versus 2,9 ± 0,6 ng/ml, respectively).

Associated with increased numbers of Th17 cells, IL-7 induced a significant increase in IL-17 production after CII restimulation (unstimulated versus CII stimulated; 0,2 ± 0,03 versus 0,6 ± 0,3 ng/ml, respectively, in the PBS-treated mice versus 0,2 ± 0,1 versus 1,4 ± 0,4 ng/ml in the IL-7 group). IL-4 concentrations were low and, in most cases, below the lowest detectable level in the supernatants of unstimulated and CII-stimulated cells from both PBS-treated mice and IL-7-treated mice (Figure 5C).

### IL-7 increases cytokines associated with T-cell activation, vascular activation, chemotaxis, and tissue destruction

**Figure 6:**
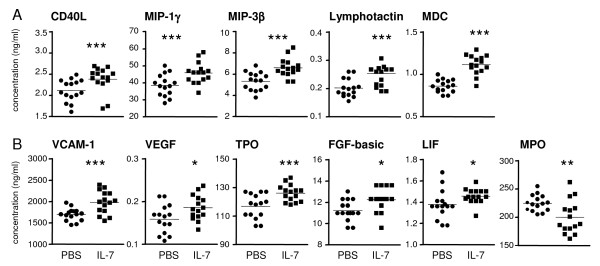
**Interleukin (IL)-7 increases mediators indicative of proinflammatory activity**. Serum concentrations of soluble CD40 ligand (CD40L), indicative of T-cell activation, were increased by IL-7 compared with phosphate-buffered saline (PBS). Chemokines facilitating chemotaxis and activation of T cells (macrophage inflammatory protein (MIP)-1a, MIP-3b, and lymphotactin) and neutrophils (macrophage-derived chemokine, MDC) were increased by IL-7. Mediators associated with vascular activation and angiogenesis (vascular cell adhesion molecule, VCAM-1; vascular endothelial cell growth factor, VEGF; thrombopoietin, TPO); and bone (leukemia inhibitory factor, LIF) and tissue (fibroblast growth factor, FGF-basic) remodeling were increased by IL-7. Neutrophil activity, indicated by myeloperoxidase (MPO) levels, was significantly reduced. Statistically significant differences of *P < 0.05, **P < 0.01, and ***P < 0.005.

The immunomodulatory effect of IL-7 on mediators indicative of activation of T cells, monocytes/macrophages, and neutrophils was analyzed with multicytokine analysis of serum. T-cell cytokines, indicative of T-cell activation (IFN-γ, IL-4, IL-17, and IL-2) were undetectable. However, concentrations of soluble CD40 ligand (sCD40L), also indicative of T-cell activation, and chemokines facilitating chemotaxis and activation of T cells and neutrophils (macrophage inflammatory protein (MIP)-1γ, MIP3β, and lymphotactin, MDC) were increased by IL-7 as compared with PBS (Figure 6A).

Concentrations of cytokines associated with monocyte/macrophage activation, such as IL-1α, IL-1β, IL-5, IL-6, IL-10, IL-18, KC, and chemokines like granulocyte chemotactic protein-2, MCP-1, MCP-3, MCP-5, MIP-1α, MIP-2, and eotaxin were detectable but were not significantly altered by IL-7 treatment (data not shown). In addition, M-CSF (macrophage-colony-stimulating factor), IgA, and CD40 were not significantly altered. TNF-α was undetectable in all groups. Opposed to increased inflammatory mediators, myeloperoxidase (MPO), an enzyme indicative of granulocyte activity, was significantly decreased with IL-7 treatment (Figure 6B).

In addition to these immunomodulatory cytokines (Figure 6A), IL-7 treatment significantly increased concentrations of mediators indicative of vascular activation and angiogenesis, such as vascular cell adhesion molecule-1 (VCAM-1), vascular endothelial cell growth factor (VEGF), and thrombopoietin (TPO) (Figure 6B). Also, cytokines associated with tissue growth and bone remodeling, fibroblast growth factor (FGF)-basic and leukemia inhibitory factor (LIF), were significantly increased by IL-7. In addition, concentrations of mediators associated with tissue growth and destruction, epidermal growth factor, matrix metalloproteinase (MMP)-9, oncostatin M, tissue inhibitor of MMP-1, and tissue factor, were not significantly changed by IL-7. The acute-phase reactant, von Willibrand factor, was increased by IL-7 (*P *< 0.05, data not shown). Acute-phase reactants such as C-reactive protein and serum amyloid P were not significantly changed.

## Discussion

This study demonstrates that IL-7 strongly increases the severity of arthritis and joint destruction in mice. Administration of IL-7 augmented radiology-assessed joint destruction, consistent with IL-7-increased intensity of cell infiltrates, bone erosions, cartilage damage, and osteoclast activity. This was associated with expansion of total splenic cell number, CD4^+ ^T cells, CD8^+ ^T cells, CD19^+ ^B cells, and germinal center B cells. IL-7 expanded circulating central and effector memory T cells as well as Th1, Th2 and Th17 cell numbers. Furthermore, collagen type II-driven activation of draining lymph node cells was associated with increased cytokines indicative of Th1 and Th17, but not Th2 activation. Finally, activation of T and B cells was associated with increases in proinflammatory cytokines and tissue-destructive mediators.

In RA patients, IL-7R^bright ^T cells are highly proliferative and largely lack Foxp3 expression, compared with poorly proliferating IL-7R^low/-^(FoxP3^high^) T cells, indicating that IL-7R^+ ^T cells in particular may promote immune activation [11]. In addition, blockade of IL-7R-mediated immune activation by soluble human IL-7 receptor inhibited IFN-γ production (indicative of Th1 cell activity) in mononuclear cells from RA patients [11]. Furthermore, IL-7 induces IL-17 production by mononuclear cells from RA patients. Th1 and Th17 activity in experimental arthritis promote joint destruction [21,22]. Consistent with these findings, we demonstrated that increased joint destruction induced by IL-7 is associated with increased antigen-driven Th1 and Th17 cell activity of draining lymph node cells.

At a systemic level, IL-7 also increased Th2 cell numbers. In contrast, antigen activation of draining lymph node cells that contain reactive T cells derived from a site of inflammation only resulted in limited amounts of IL-4, which was reduced to undetectable levels on IL-7 treatment. These data suggest that, although as a consequence of the homeostatic potential of IL-7, CD4^+ ^T cells producing IL-4 may be increased systemically, this may not result in significant intraarticular IL-4 production, contributing to the predominance of Th1 and Th17 activity. The local predominance of Th1 and Th17 over Th2 activity in joints of RA patients is in line with these data [23,24]. Supporting these findings, IL-7R blockade was associated with reduced IFN-γ and IL-17 levels and with reduced arthritis severity and immunopathology [19].

IL-7 has significant effects on T-cell numbers in many experimental studies and in humans, and has been shown to increase B-cell numbers profoundly in mice in contrast with humans [25,26]. The stimulatory effects of IL-7 on CIA in this study, associated with expansion of central and effector memory CD4^+ ^T cells, and increased B cells and germinal center B cells, are in line with these results. Although we did not analyze B-cell activation markers in our study, the significant increase in numbers of germinal center B cells, which are potent antibody-producing cells, suggests that IL-7 facilitates B-cell activation either direct or indirect via T cells or other cell types and, through this, plays a significant role in the IL-7-induced increased immunopathology. In line with this, we demonstrated that IL-7 induces T-cell- and monocyte-dependent B-cell activation in a human *in vitro *model [27].

Decreased MPO might reflect reduced circulating neutrophil numbers as a consequence of granulocyte migration to the site of inflammation, but this remains to be demonstrated. In line with this, we demonstrated that local levels of MPO were abundantly present and strongly reduced with IL-7R blockade, indicating that IL-7R-induced T-cell activation and increased arthritis are associated with increased granulocyte activity in the inflamed joints [19].

In support of the T-cell activation seen in this study, serum concentrations of CD40L, indicative of T-cell activation, were increased by IL-7. In addition to inducing T-cell-related cytokines, IL-7 can induce other proinflammatory cytokines, largely produced by human monocytes, including TNF-α, IL-1β, IP-10, MIG, MIP-1γ, TARC, and IL-8 [8,10,12,14-16]. In line with these proinflammatory capacities, we demonstrated that IL-7 administration in CIA mice significantly upregulates chemokines that are capable of facilitating migration of lymphocytes (MIP1γ, MIP-3β, and lymphotactin), monocytes/macrophages (MCP-5), and granulocytes (MDC). The immune-stimulatory effect of IL-7 was also reflected by a significant increase in von Willibrand factor and mediators indicative of endothelial cell adhesion and angiogenesis: VCAM-1, VEGF, and TPO. The latter two have been described as very important in initiation of synovial hyperplasia in RA [28,29]. Together, this strongly suggests that IL-7 facilitates arthritis by increasing chemotaxis of leukocytes, together with enhancement of vascular permeability and neovascularization, eventually leading to increased synovitis and tissue destruction.

IL-7-induced activation of human effector CD4^+ ^T cells upregulates expression and production of receptor activator of nuclear factor (NF)-κB ligand (RANKL), a key factor in osteoclastogenesis regulation [30,31]. Supporting the described capacity of IL-7 to increase joint destruction, the present study showed that IL-7 increased leukemia inhibitory factor (LIF) and FGF-basic. LIF is an IL-6-like cytokine, involved in regulation of bone remodeling and bone cell function by stimulation of RANKL [32,33]. FGF-basic induces RANKL expression and osteoclast maturation [34]. These data coincide with T cell-dependent osteoclast activity/formation, leading to osteopenia and increased bone resorption in IL-7-overexpressing transgenic mice [35].

Increased loss of joint space, indicating cartilage destruction, was observed on treatment with IL-7. This may result from a local IL-7-induced increase in proinflammatory mediators that induce cartilage destruction, such as TNF-α and oncostatin M. IL-7R blockade has been demonstrated to reduce local expression of such cytokines, thereby preventing cartilage destruction [19]. Unfortunately, in the present study, mediators such as TNF-α and oncostatin M could not be detected in the serum of arthritic mice. However, IL-7 administration in CIA did increase concentrations of FGF-basic, which has been suggested to be involved in cartilage degradation, mostly through production of MMPs. In addition, cartilage damage may result from direct effects of IL-7 on articular chondrocytes. Chondrocytes were found to express IL-7R and respond to IL-7 stimulation with production of MMP-13 and with proteoglycan release from cartilage explants [36]. Furthermore, increased IL-7 expression has been detected in chondrocytes from RA patients [37]. Although in the present study, a direct effect of IL-7 on cartilage could not be demonstrated, this study demonstrates IL-7-induced cartilage degradation in addition to bone destruction.

## Conclusions

Our results demonstrated that IL-7 enhances joint inflammation and joint damage in CIA. This was associated with a selective expansion of memory T cells, a specific increase in Th1 and Th17 cells, as well as mediators that promote inflammation and tissue destruction. Together, this indicates that blockade of IL-7 or the IL-7R, preventing IL-7R-induced immune activation by IL-7, might be successful therapeutic strategies to inhibit T-cell-driven inflammation and joint destruction in arthritis. Because IL-7 increases T-cell activation, arthritis, and joint destruction in the current autoimmune model, this also suggests that administration of IL-7 to humans should be carefully evaluated [38,39].

## Abbreviations

CFA: complete Freund adjuvant; CIA: collagen-induced arthritis; CII: chicken collagen type II; DN: double negative; DP: double positive; EGF: epidermal growth factor; FGF: fibroblast growth factor; HE: hematoxylin-eosin; IACUC: Institutional Animal Care and Use Committee; IFA: incomplete Freund adjuvant; IFN: interferon; IL: interleukin; IL-7Ra: interleukin-7 receptor-α chain; LIF: leukemia inhibitory factor; LN: lymph node; LNs: lymph node cells; MAP: multianalyte profiling; MCP: monocyte chemotactic protein; M-CSF: macrophage-colony-stimulating factor; MDC: macrophage-derived chemokine; MIP: macrophage inflammatory protein; MMP: matrix metalloproteinase; MPO: myeloperoxidase; PB: peripheral blood; RA: rheumatoid arthritis; RANKL: receptor activator of NF-κB ligand; sCD40L: soluble CD40 ligand; SF: synovial fluid; TIMP: tissue inhibitor of MMP; TNF: tumor necrosis factor; TPO: thrombopoietin; TSLP: thymic stromal lymphopoietin; TSLPR: thymic stromal lymphopoietin receptor; VCAM: vascular cell-adhesion molecule; VEGF: vascular endothelial cell growth factor.

## Competing interests

CW owns stock or stock options in Amgen Inc. SH, JB, FL, and JvanR have no competing interests to declare.

## Authors' contributions

All authors were involved in drafting the article or revising it critically for important intellectual content, and all authors approved the final version to be published. JR had full access to all of the data in the study and takes responsibility for the integrity of the data and the accuracy of the data analysis. Study conception and design were performed by SH, CW, JB, FL, and JR; acquisition of data was done by SH and CW, and analysis and interpretation of data, by SH, CW, and JR.
